# Supplementary material for: Retinal Proteomic Alterations and Combined Transcriptomic-Proteomic Analysis in the Early Stages of Progression of a Mouse Model of X-Linked Retinoschisis
Source: Cells. 2022 Jul 8;11(14):2150. doi: 10.3390/cells11142150 (PMC9321393; doi:10.3390/cells11142150)
Supplement: Supplementary file 1 [file cells-11-02150-s001.zip › Figures S1.pdf]

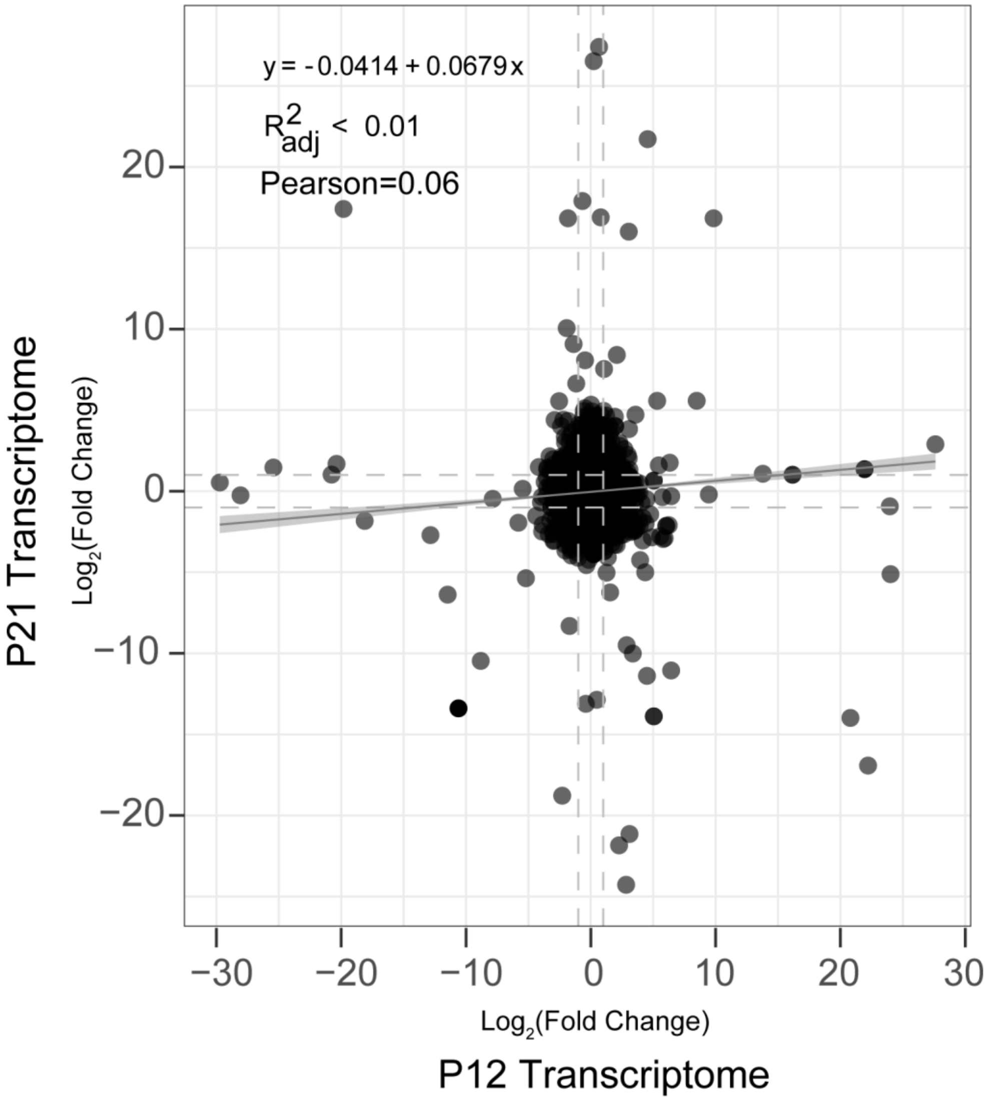

Figure S1. Correlation analysis of the altered transcriptome at P12 and P15. The data were downloaded from National Center for Biotechnology Information Gene Expression Omnibus (https://ncbi.nlm.nih.gov/geo/): GSE153874). Those genes quantified in all samples (P12_WT, P12 Rs1 KO, P21_WT, and P21_Rs1 KO) were identified as quantified genes. In total, 26,005 quantified genes (remove duplicate values) were used for correlation analysis.
